# Moxibustion Treatment for Knee Osteoarthritis: A Systematic Review and Meta-Analysis

**DOI:** 10.1097/MD.0000000000003244

**Published:** 2016-04-08

**Authors:** Ang Li, Zhi-Jian Wei, Yi Liu, Bo Li, Xing Guo, Shi-Qing Feng

**Affiliations:** From the Department of Orthopedics, Tianjin Medical University General Hospital, Heping District, Tianjin, China.

## Abstract

To determine whether the administration of moxibustion is an effective treatment for knee osteoarthritis (KOA).

We conducted a search of relevant articles using Medline, EMBASE, the Web of Science, and the Cochrane Library published before October 2015. The Western Ontario and McMaster Universities’ Osteoarthritis Index (WOMAC scale) and the short form 36 questionnaire (SF-36 scale) were assessed. Evidence grading was evaluated according to the Grading of Recommendations, Assessment, Development and Evaluation system.

Four studies containing 746 participants fulfilled the inclusion criteria in the final analysis. In terms of quality of life (QOL), the meta-analysis of 2 randomized clinical trials (RCTs) showed significantly effects of moxibustion only in bodily pain (BP) compared with those in the control group (n = 348; weighted mean difference [WMD], 4.36; 95% confidence intervals [CIs], 2.27–6.44; *P* < 0.0001; heterogeneity: χ^2^ = 1.53, *P* = 0.22, *I*^2^ = 34%) in all of the subcategories of the SF-36 scale, with moderate quality. The meta-analysis of the 2 included trials showed that there was not a statistically significant difference in the pain or function subscale for the WOMAC scale when the 2 groups were compared (n = 322; WMD, 17.63; 95% CI, −23.15–58.41; *P* = 0.40; heterogeneity: χ^2^ = 19.42, *P* < 0.0001, *I*^2^ = 95%), with low or moderate quality separately.

The administration of moxibustion can to some extent alleviate the symptoms of KOA. More rigorous, randomized controlled trials are required in the future.

## Abstract 2

Supplemental Digital Content is available in the text

## INTRODUCTION

Knee osteoarthritis (KOA), known as severe degenerative arthritis, is common in middle-aged and elderly people. It causes pain and dysfunction that greatly reduce the patients’ quality of life (QOL).^1^ An earlier study demonstrated that Chinese people more than 60 years of ages hared a high incidence (19.4%) of doctor-diagnosed and symptomatic KOA.^2^ This number is expected to rise in the future because of increases in the obese population and in life expectancy.^3^ However, the nonsurgical treatment of KOA has not changed in recent decades, and the symptomatic treatment based on pharmacological therapy was still the prevalent as well as the preferred mode of treatment.^4^ Unsurprizingly, a prolonged therapy with medication would lead to undesired side effects, such as renal and hepatic toxicity, gastrointestinal bleeding, and ulcers.^5^

Moxibustion is a traditional oriental medicine that stimulates acupuncture points through heat generated by a flaming moxa wool, the herb artemisia vulgaris. It has frequently been recommended to treat a variety of illnesses as an adjuvant therapy, with or without acupuncture, such as malposition, colitis, muscle strain, urinary incontinence, soft-tissue injury, postlaminectomy pain, asthma, reactive arthritis, fibromyalgia, and conditions related to aging.^6^ KOA is the 3rd common indication.^7,8^ Moxibustion is widely utilized for the treatment of KOA in Asian countries.^6^ Currently, moxibustion treatments can be categorized as traditional moxibustion, drug moxibustion, and modern moxibustion.^9^ The present meta-analysis focused on traditional moxibustion, including both its direct or indirect application (i.e., whether there is contact with the skin). Currently, there are many animal experiments and reports about the clinical positive efficacy of moxibustion for the treatment of arthritis and pain;^10–12^ however, there is no rigorous evidence supporting this conclusion. The aim of the present meta-analysis was to evaluate the efficacy and safety of moxibustion in the management of patients with KOA.

## METHODS

### Literature Search

We searched the following electronic databases: Medline, EMBASE, the Web of Science, and the Cochrane Library. The search language was restricted to English. The search strategy was based on a combination of 2 concepts adjusted to each database. Concept 1 included all of the terms for KOA, and concept 2 included the terms for moxibustion. The Boolean operator AND was used to link the 2 concepts. The bibliographies of the included studies and dissertations were searched for additional publications. Additionally, relevant journals as well as files were manually searched and thoroughly read; we only accepted 1 set of data on the same topic in the event of multiple publication bias. All of the eligible studies were identified by 2 independent authors (AL, ZJW), and any disagreements were settled by consensus or consultation with a 3rd author (BL).

### Study Selection

We included these studies into the meta-analysis if they fulfilled all of the following inclusion criteria: trials written in English had to be properly randomized; moxibustion was used as the sole intervention measure or was combined with another standard treatment for KOA, such as conventional medication or physiotherapy; and both groups can received the same foundation therapy. The exclusion criteria were as follows: the evaluation of the efficacy of moxibustion was absent in the studies; there was crossover in principle between the intervention in the control groups and moxibustion; the study was related to the comparison of the 2 types of moxibustion; and case reports, editorials, experimental studies, conference articles, non-English studies, and other studies that failed to provide detailed results were excluded.

### Quality Assessment

Cochrane collaboration tool for assessing risk of bias was used to evaluate the methodological quality of the included trials.^13^ This tool focuses on the internal validity of the trial and assessment of risk of possible bias in different phases of the trial. The details are as follows: random sequence generation, allocation concealment, blinding of outcome assessment, blinding of participants and personnel, incomplete outcome data, selective reporting, and other bias. Each item was classified according to a high, low, or unclear risk of bias that is represented as high (H), low (L), and unclear (U), respectively. All of the assessments were conducted by 2 independent reviewers (AL, XG). Any controversies were settled by consensus or discussion with a 3rd author (BL).

### Data Extraction

The relevant data from the eligible papers were double extracted by 2 authors (AL, YL) according to a predefined standardized protocol. Pertinent details included: author information, year, sample size, country, diagnostic criteria, patient's age, outcome measures, intervention, and control regimen. Any discrepancies were resolved by consensus. When inadequate information existed in the studies, contacting the 1st authors to obtain and clarify the relevant data were essential as specified by the standardized protocol.

### Outcome Assessment

The Western Ontario and McMaster Universities’ Osteoarthritis Index (WOMAC scale)^14^ and the short form 36 questionnaire (SF-36 scale)^15^ expressed as the standardized mean difference were used to analyze the summary estimates. The WOMAC scale, which includes ratings of pain, stiffness, and function, was used to evaluate the disability level of the patients with KOA. The SF-36 scale was used to assess the patients’ QOL and it covers 8 dimensions: physical functioning (PF), physical role functioning, bodily pain (BP), general health (GH), vitality (VT), social role functionality (SF), emotional role functionality (RE), and mental health.

### Statistical Analysis

The Review Manager Software Package (RevMan Version 5.2, The Cochrane Collaboration, Copenhagen, 2014) was used to generate forest plots. The overall effect of moxibustion treatment was calculated as the weighted average of the inverse variance adjusted individual effects and 95% confidence intervals (95% CIs). The statistical heterogeneity among the individual studies was evaluated based on Cochrane Q test and the *I*^2^ index,^16^ and the statistical heterogeneity was confirmed if *I*^2^ was >75% and *P* < 0.10.^17^ A variance-based fixed effect model was applied to calculate the pooled effect; otherwise, a random effect model was used in the presence of statistically significant heterogeneity.^18^ If appropriate, the heterogeneity was identified and explained using a subgroup analysis.^16^ Evidence grading was evaluated according to the Grading of Recommendations, Assessment, Development, and Evaluation system.^19^

### Ethical Statement

As all analyses were grounded on previously published studies, ethical approval was not necessary.

## RESULTS

### Search Results

**FIGURE 1:**
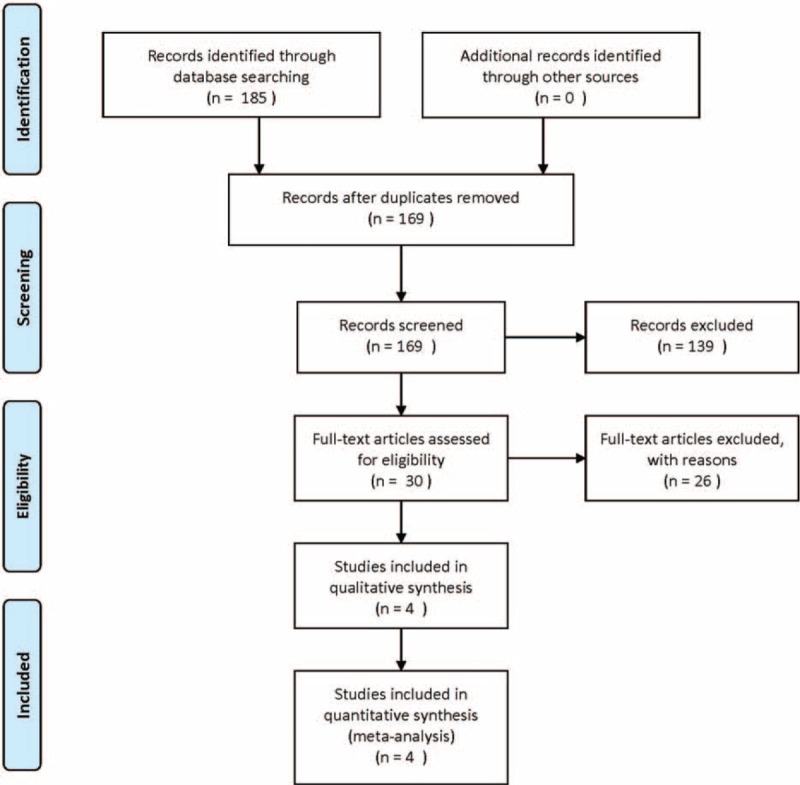
Flow Diagram of the Study.

The initial literature search yielded a total of 185 articles. After duplicate checking and title and abstract screening, 30 publications met the inclusion criteria, and the full text of all 30 articles was available. Among these articles, 3 articles were excluded because intervention was not the sole moxibustion but was combined with acupuncture or medication; 4 articles were excluded because of a comparison of heat-sensitive moxibustion (HSM) and conventional moxibustion in the treatment of KOA; 2 articles were excluded because they were study protocols; and 17 articles were excluded because they were not written in English. Moreover, a manual search of relevant references did not identify additional studies. Finally, 4 intermediate- to high-quality studies^20–23^ were eligible for inclusion in the present meta-analysis (Figure 1).

### Participants and Study Characteristics

Table 1 shows the characteristics of the eligible trials. Of the 4 citations included,^20–23^ a total of 746 participants (370 moxibustion, 376 control) divided into 8 groups (4 moxibustion, 4 control) were recruited in the final analysis. All of the studies were multi-center trials. Overall, 3 trials were performed in China, and 1 was performed in Korea.^23^ The moxibustion treatments in 3 randomized clinical trials (RCTs)^20–22^ were based on the traditional Chinese medicine theory as the guide for the implementation procedures. The points were chosen according to the traditional Korean medicine in the other RCT.^23^ Assessing the expectation regarding the effectiveness of moxibustion on KOA was conducted in 1 RCT.^23^ The patients in the studies were middle-aged and elderly; the average age ranged from 38 to 70 years. The mean duration of follow-up ranged from 3 to 28 weeks. The intervention typesin the control groups were sham moxibustion in 2 studies,^20,21^ intraarticular sodium hyaluronate in 1 study,^22^ and usual care (UC) in 1 study.^23^ One RCT tested the positive effects of moxibustion on KOA, utilizing the following 3 randomized groups: HSM group, conventional moxibustion group, and intraarticular sodium hyaluronate control group.^22^ The HSM group was removed from this meta-analysis. KOA in patients retrieved from the articles was confirmed according to the American College of Rheumatology criteria in 3 trials,^20,21,23^ and the criteria of the Guiding Principles of Clinical Research on New Drugs were used to diagnosis KOA in 1 study.^22^ Two RCTs calculated the appropriate sample capacity before conducting the trials according to the previous pilot study.^22,23^ Three RCTs reported ethical approval of the study protocol from their Institutional Review Boards before the study enrollment of the 1st participant.^20,21,23^

**TABLE 1:**
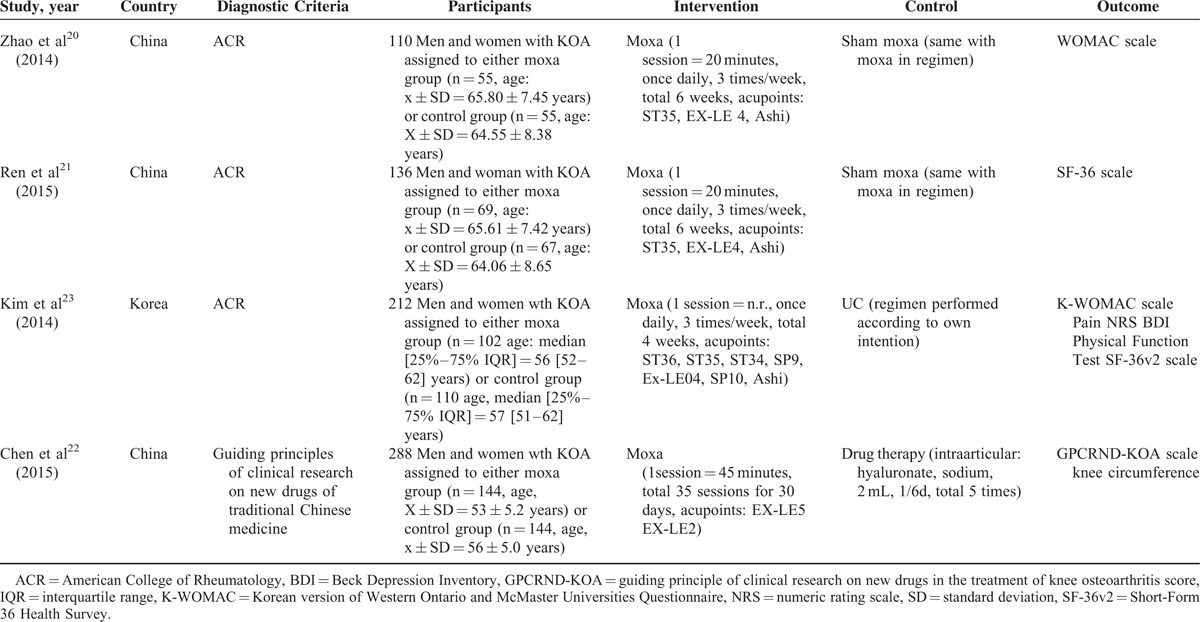
Summary of Characteristics in Studies Included

### Risk of Bias Assessment

Based on the Cochrane Collaboration recommendation, 4 RCTs adopted allocation concealments, and comprehensive methodological processes and random sequence generation were reported. Assessor blinding was determined to be at a low risk of bias in all of the included studies. The participants and personnel were blinded in 2 trials;^20,21^ 1 RCT conducted an assessment of blinding effectiveness.^20^ Sufficient details of withdrawals and dropouts were described in all 4 studies. Two studies used the intention-to-treat approach in the data handling.^20,22^ One RCT reported the remedy for dropout.^21^ In the majority of the studies, whether enrollment of the participants was actually consecutive or not was unclear, so a selection bias could be completely excluded. The details of risk of bias are illustrated in Supplemental Figure 1.

### Adverse Events

Four RCTs reported relevant adverse events (AEs) in the active moxibustion group but only 1 AE in the control group; 1 study^22^ demonstrated that no AEs occurred in the study period. Otherwise, 3 RCTs^20,21,23^ noted region adverse reactions, for example, blisters, burn wounds, and skin flushing in local lesions. Two studies^21,23^ reported systemic AEs; however, only 1^23^ reported positive symptoms, that is, pruritus and fatigue. Furthermore, 1 RCT^23^ assessed the severity of each AE according to Spilker AE classification^24^ and reported a high incidence (47%) of abnormal reactions, the majority of which were second-degree burns.

### WOMAC in Patients With Moxibustion and Non-Moxibustion

**FIGURE 2:**
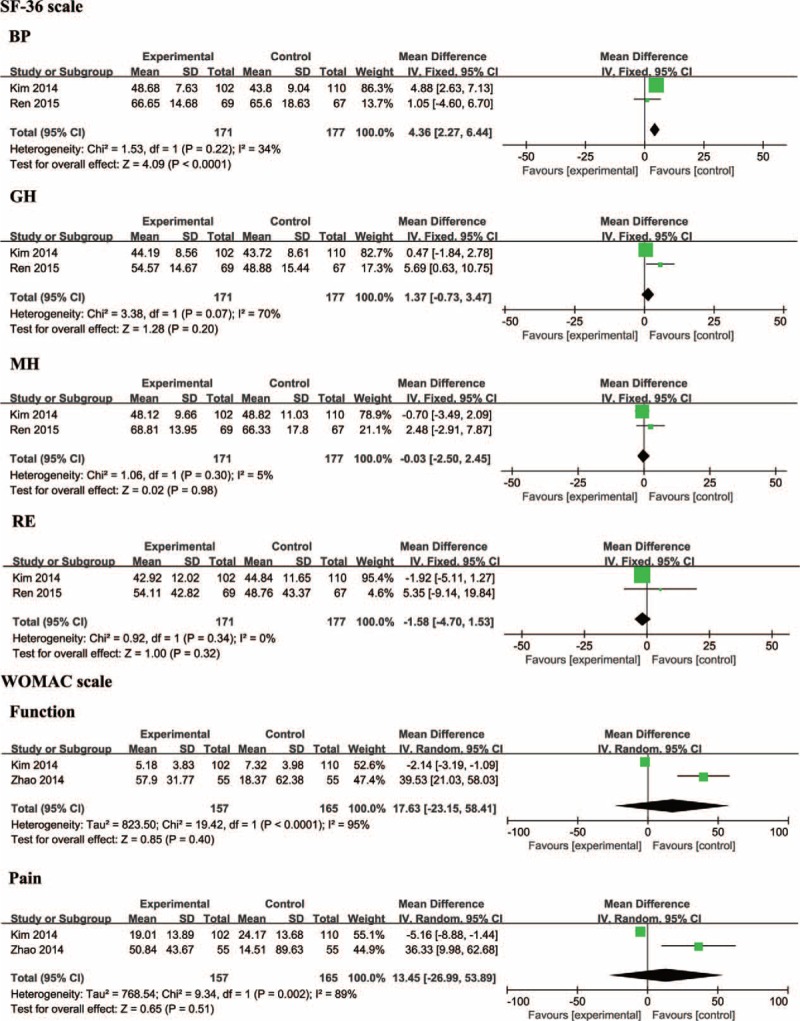
Forest Plot for Some Outcomes.

In total, 2 studies^20,23^ reported the WOMAC scores in patients with moxibustion. The control group was sham-moxibustion or UC separately. A considerable statistical heterogeneity existed in the pain scores between the 2 studies (χ^2^ = 19.42, *P* < 0.0001, *I*^2^ = 95%), and the fixed-effects model was applied for performing the data analysis. Otherwise, the meta-analysis of the 2 eligible trials showed that there was not a statistically significant difference in the pain scores (n = 322; weighted mean difference [WMD], 17.63; 95% CI −23.15–58.41; *P* = 0.40), although the participants with moxibustion statistically achieved a significantly greater improvement in the pain symptoms of WOMAC scale compared with the control groups in each study. With respect to the function score, which was similar to the pain score, the meta-analysis demonstrated that the function score in patients with moxibustion was not significantly lower, and it had a high heterogeneity (n = 322; WMD, 13.45; 95% CI, −26.99–53.89; *P* = 0.51; heterogeneity: χ^2^ = 9.34, *P* = 0.002, *I*^2^ = 89%) (Figure 2).

### SF-36 in Patients With Moxibustion and Non-Moxibustion

Two studies^21,23^ assessed the QOL in patients with KOA using the SF-36 scale. The control group was sham-moxibustion or UC separately. One RCT^21^ revealed that the participants with moxibustion experienced a considerably significant improvement in a GH score than the control group at week 6 (*P* = 0.015) and week 12 (*P* = 0.029); the same result applied to the VT score at week 12 (*P* = 0.042). The other study^23^ reported that the BP score, PF score, or SF score in the patients with moxibustion were separately higher than those scores of the patients in the control group (*P* = 0.0003 at week 5, 13; *P* = 0.0025 at week 5; and *P* = 0.0418 at week 5). The data in all of the subcategories of the SF-36 scale were quantitatively synthesized in the meta-analysis. The results showed that the BP score in the patients with moxibustion was significantly higher than that in the controls (n = 348; WMD, 4.36; 95% CI, 2.27–6.44; *P* < 0.0001; heterogeneity: χ^2^ = 1.53, *P* = 0.22, *I*^2^ = 34%). However, no difference was detected with respect to the other domains in the intergroups: PF (WMD, 0.98; 95% CI, −0.97–2.93; *P* = 0.32); mental health (WMD, −0.03; 95% CI, −2.50–2.45; *P* = 0.98); emotional role functionality (WMD, 1.58; 95% CI, −4.70–1.53; *P* = 0.30); physical role functioning (WMD, 1.13; 95% CI, −1.41–3.68; *P* = 0.38); SF (WMD, 0.61; 95%, CI −1.50–2.72; *P* = 0.57); VT (WMD, 2.92; 95% CI, 0.32–5.51; *P* = 0.03), and GH (WMD, 2.57; 95% CI, −2.44–7.59; *P* = 0.31). The data regarding the other 4 items are listed in Supplemental Figure 2.

### Evidence Grading

Evidence grading was evaluated according to the Grading of Recommendations, Assessment, Development and Evaluation system^19^ (Supplemental Table). Ten outcomes in the WOMAC scale or SF-36 scale were analyzed in this meta-analysis. The level of evidence was moderate for 8 aspects in the SF-36 scale. For the WOMAC scale, the level of evidence was low for the pain score and moderate for the function score.

## DISCUSSION

Overall, many clinical trials have tested the efficacy of moxibustion in the treatment of KOA.^3,25,26^ In fact, the results of this meta-analysis supported, to some extent, the above suggestion. However, because of the small number of trials, low consistency, and standardization of the control intervention as well as the potential bias in the existing studies, we decided not to determine the practical availability of the management of KOA due a lack of sufficient supporting evidence.

To date, there have been 3 related systematic reviews about moxibustion regarding health supervision for KOA,^8,27,28^ all of which have clarified that moxibustion was significantly beneficial to relieving pain and improving function for patients with KOA. However, nearly all of the trials regarding KOA included in the above systematic reviews had a high risk of bias and low methodological quality. The purpose of this paper was to evaluate the effects of moxibustion on symptom management for patients with KOA by not only adding new clinical trials but also simultaneously improving the research quality of the studies. All of the studies included were characteristic of multicenter, randomized controlled trials, which guaranteed its external validity. An appropriate randomization sequence was generated in all of the trials included in this present analysis. Inadequate sequence generation in randomization studies tended to yield exaggerated treatment effects.^29^

Additionally, some RCTs^22,23^ included here employed various core outcome domains, such as functional performance tests (6-minute walk test); some objective and disease-specific parameters (knee circumference) quantify the results rather than simply evaluate the patients’ ratings of improvement and pain intensity used in other literature.^30^ To date, the SF-36 scale is the most widely used QOL measure in patients with health-related conditions.^15^ Initially developed in 1982, the WOMAC scale targeted patients with knee and hip osteoarthritis and has been validated in >80 language translations.^14^ The patient's change in pain-related experience might be more incisively shown by the WOMAC pain subscale and the SF-36 BP scale, because it was reported that the pain-numeric rating scale was too superficial and simple to evaluate the complexity of a patient's pain experience.^31^

As no significant difference existed in between groups (Guiding Principles of Clinical Research on New Drugs-KOA scores, *P* = 0.130; knee circumference, *P* = 0.141), sodium hyaluronate intraarticular injection as a conventional treatment for KOA had been validated and widely acknowledged by the general,^32^ which indirectly identified that moxibustion played an important role in alleviating the symptoms in adults with KOA. Likewise, UC is the same as the above mentioned,^33^ but it is more straightforward because of a moderate effect size observed at 5 weeks (WOMAC score, *P* = 0.0477) and 13 weeks (WOMAC score, *P* = 0.0518) between groups. Additionally, each component of UC was based on previous evidence of benefits in the treatment management of KOA.^34^ In another study,^22^ no significant difference existed in the outcomes between conventional moxibustion with conventional medication groups in patients with KOA at each time point, indicating the effect of moxibustion in KOA.

At present, the AEs announced in the published literature concerning moxibustion therapy primarily involve burns, allergies, infections, nausea, loose lower eyelids, ectropion, death, and so on.^6,27^ However, the issue of whether moxibustion-induced burns are, in fact, an AE is still controversial.^27^ In Chinese traditional moxibustion, also known as scarring moxibustion, local minor burns, scarring, and purulence during treatment were taken for granted because many of the components take effect after entering the human body through burn-damaged skin.^23,35^ Additionally, in term of burns, some patients accepted these scars as the natural conclusion of moxibustion therapy. When weighing the pros and cons, it was reported that the appropriate distance for indirect moxibustion seems to be 3 to 4 cm.^36,37^ Furthermore, how much causal association between some AEs and moxibustion is still unclear. Certain factors, such as the duration, position, distance between moxa sticks and skin, the patient's conditions, physicians’ proficiency, stimulations from smoke, and even the treatment environment, and so on, can affect the safety of moxibustion.^20,38^

If the efficacy of moxibustion as a treatment for KOA is acknowledged, the underlying mechanism would draw much attention. Interests in research regarding the underlying mechanisms of moxibustion have grown in recent decades.^39,40^ Currently, the therapeutic effects of moxibustion are generally believed to be derived from radiation effects, thermal effects, and the pharmacological actions of moxa combustion.^41^ The ingredients of moxa smoke are complex, consisting of terpene compounds, aliphatic hydrocarbons, alcohols, aromatic hydrocarbons, and their oxides.^25,42^ Nevertheless, the exhaustive understanding of the mechanisms of moxibustion therapy is still limited. It was previously reported that the sensation of heat as the foundation of moxibustion treatment is important.^25^ According to the traditional Chinese medicine theory, moxibustion is analogous to acupuncture in principle. Although it is more superficial, its effect on the sensory nerves is well known as “Zhen Jiu” in ancient China, and a mass of Chinese literatures or systematic reviews reported heat's responsibility for symptom management in KOA.^43–45^ The synergistic effects of heat derived from moxibustion on the stimulation of some specific acupoints might be alikely mechanism. Today, the primary speculation about moxibustion is that it acts through the local or system neural network and releases some neurotransmission, such as opioidergics, beta endorphins, and adenosine triphosphate.^46,47^ Moreover, it was reported that moxibustion could modulate the inflammatory reactions through the degranulation of local mastocytes and activation of thermoreceptors and further normalize the immune system in a KOA rat model.^48^ Many studies have revealed that moxibustion could ease the nociceptive painful reception and improve the force of a rat's limb tread through the regulatory mechanism of some signaling molecules, transcription factors, and even some mRNA, such as nitric oxide, signal transducer and activator of transcription 1, suppressor of cytokine signaling, c-Fos, transforming growth factor-β, insulin-like growth factor-I, and neuronal nitric oxide synthase.^39,42,49^ It was reported that the toll-like receptor-4 – myeloid differentiation factor 88 – nuclear factor kappa B signal transduction pathway was related to the changes in the knee-joint synovial tissue in rats with rheumatic arthritis.^50^ However, so far, all of these theories are little more than speculation.

Given the special feature of KOA, an active control was required to relive the patient's condition, and it was impossible to blind acupuncturists to the treatment because of the nature of the intervention.^51^ The sham moxibustion device, which is essential for differentiating the specific from the non-specific treatment effects, did produce heat but to a lesser extent than that of the true application. Although the sham device resembles the real one in appearance, and the reliability was previously examined and validated by Zhao et al,^52^ controversies still existed and there was some doubt regarding the availability of a double-blinded, randomized controlled trial in moxibustion research.^53^ Kim et al^54^ reported a sham moxibustion device that was conditioned as much as possible to the minimum temperature; however, it could reach a heat level of 39 °C. However, some physiological effect caused by this lower temperature of heat cannot be precluded. Additionally, compared with the verum, the nonspecific effects of the sham moxibustion might come from preventing heat stimulation on acupoints or affecting other areas beyond the acupuncture points. Moreover, moxibustion is primarily used only in the eastern countries but not in Europe, and the patients possess high expectations in these studies.^51^ As moxibustion can bring about such positive placebo effects, the consequence of moxibustion plus a standard treatment in comparison to only the administration of a standard treatment is most likely to be a significant positive result. Hence, to realize that the patients were blinded, 2 RCTs^20,23^ recruited the patients who were naive to moxibustion and had never received moxibustion therapy until the study began, which might lead to a selection bias.

The limitations of this systematic review involve restrictions on the publication language, uniformity of the control group or moxibustion program and a small number of included RCTs. Some literatures or guidelines have well documented the distorting effects of location bias and publication bias on the systematic reviews and meta-analyses.^55–57^ In the present review, the retrieval language limited to English would generate a sampling bias, and because the majority of the trials are from China, some potential uncertainty concerning positive evidence for moxibustion might, to some extent, exist. Moreover, moxibustion is applied in clinical practice together with a high heterogeneity in the stimulating process, original materials, duration, frequency, selection of acupoints, etc.^28^ The empirical evidence from previous research has suggested that the therapeutic effect of moxibustion depends on the following 3 key factors: dose, location, and sensation. The effects of moxibustion may vary across any change in each factor. The high-level evidence requires wide strictness and consistency to support.

## CONCLUSION

To a certain extent, moxibustion is likely to manage the symptoms and improve the QOL among the selected patients with KOA. Additionally, more well-designed, rigorous, randomized controlled trials on this subject are required to confirm the outcome validity of this meta-analysis.

## Supplementary Material

Supplemental Digital Content
