# Supplementary material for: Moxibustion Treatment for Knee Osteoarthritis: A Systematic Review and Meta-Analysis
Source: Medicine (Baltimore). 2016 Apr 8;95(14):e3244. doi: 10.1097/MD.0000000000003244 (PMC4998779; doi:10.1097/MD.0000000000003244)

| **Effectiveness of moxibustion for KOA** | | | | | | |
| --- | --- | --- | --- | --- | --- | --- |
| **Patient or population:** patients with KOA **Settings:** hospital **Intervention:** effectiveness of moxibustion | | | | | | |
| **Outcomes** | **Illustrative comparative risks* (95% CI)** | | **Relative effect (95% CI)** | **No of Participants (studies)** | **Quality of the evidence (GRADE)** | **Comments** |
|  | Assumed risk | Corresponding risk |  |  |  |  |
|  | **Control** | **Effectiveness of moxibustion** |  |  |  |  |
| **Pain** WOMAC scale. Scale from: 0 to 50. Follow-up: mean 12.5 weeks | The mean Pain in the control groups was **12.56** | The mean Pain in the intervention groups was **17.63 higher** (23.15 lower to 58.41 higher) |  | 322 (2 studies) | ⊕⊕⊝⊝ **low** |  |
| **Function** WOMAC scale. Scale from: 0 to 170. Follow-up: mean 12.5 weeks | The mean Function in the control groups was **19.83** | The mean Function in the intervention groups was **13.45 higher** (26.99 lower to 53.89 higher) |  | 322 (2 studies) | ⊕⊕⊕⊝ **moderate** |  |
| **PF** SF-36. Scale from: 0 to 100. Follow-up: mean 12.5 weeks | The mean PF in the control groups was **42.39** | The mean PH in the intervention groups was **1.17 higher** (0.9 lower to 3.24 higher) |  | 281 (2 studies) | ⊕⊕⊕⊝ **moderate** |  |
| **MH** SF-36. Scale from: 0 to 100. Follow-up: mean 12.5 weeks | The mean MH in the control groups was **52.51** | The mean MH in the intervention groups was **0.03 lower** (2.5 lower to 2.45 higher) |  | 348 (2 studies) | ⊕⊕⊕⊝ **moderate** |  |
| **SF** SF-36. Scale from: 0 to 100. Follow-up: mean 12.5 weeks | The mean SF in the control groups was **52.76** | The mean SF in the intervention groups was **0.61 higher** (1.5 lower to 2.72 higher) |  | 348 (2 studies) | ⊕⊕⊕⊝ **moderate** |  |
| **VT** SF-36. Scale from: 0 to 100. Follow-up: mean 12.5 weeks | The mean VT in the control groups was **49.89** | The mean VT in the intervention groups was **2.92 higher** (0.32 to 5.51 higher) |  | 348 (2 studies) | ⊕⊕⊕⊝ **moderate** |  |
| **RP** SF-36. Scale from: 0 to 100. Follow-up: mean 12.5 weeks | The mean RP in the control groups was **44.21** | The mean PR in the intervention groups was **1.13 higher** (1.41 lower to 3.68 higher) |  | 348 (2 studies) | ⊕⊕⊕⊝ **moderate** |  |
| **RE** SF-36. Scale from: 0 to 100. Follow-up: mean 12.5 weeks | The mean RE in the control groups was **45.02** | The mean RE in the intervention groups was **1.58 lower** (4.7 lower to 1.53 higher) |  | 348 (2 studies) | ⊕⊕⊕⊝ **moderate** |  |
| **GH** SF-36. Scale from: 0 to 100. Follow-up: mean 12.5 weeks | The mean GH in the control groups was **44.61** | The mean GH in the intervention groups was **2.57 higher** (2.44 lower to 7.59 higher) |  | 348 (2 studies) | ⊕⊕⊕⊝ **moderate** |  |
| **BP** SF-36. Scale from: 0 to 100. Follow-up: mean 12.5 weeks | The mean BP in the control groups was **46.79** | The mean BP in the intervention groups was **4.36 higher** (2.27 to 6.44 higher) |  | 348 (2 studies) | ⊕⊕⊕⊝ **moderate** |  |
| *The basis for the **assumed risk** (e.g. the median control group risk across studies) is provided in footnotes. The **corresponding risk** (and its 95% confidence interval) is based on the assumed risk in the comparison group and the **relative effect** of the intervention (and its 95% CI). **CI:** Confidence interval; | | | | | | |
| GRADE Working Group grades of evidence **High quality:** Further research is very unlikely to change our confidence in the estimate of effect.  **Moderate quality:** Further research is likely to have an important impact on our confidence in the estimate of effect and may change the estimate. **Low quality:** Further research is very likely to have an important impact on our confidence in the estimate of effect and is likely to change the estimate. **Very low quality:** We are very uncertain about the estimate. | | | | | | |
|  |  |  |  |  |  |  |

Supplemental Table: Summary of the findings

Supplemental Figure 1: Risk of bias summary and grap


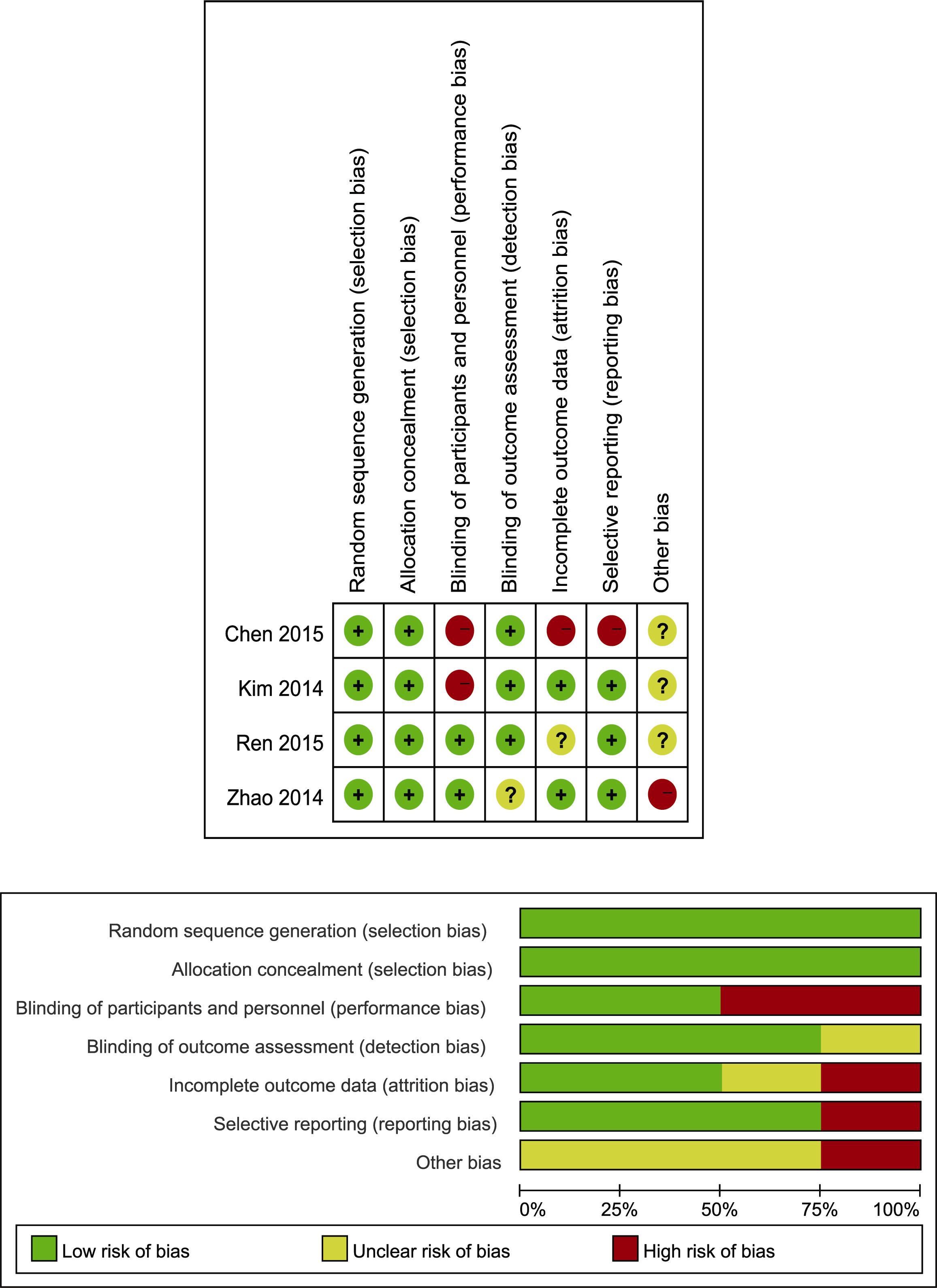


Supplemental Figure 2: Forest plot for other outcomes


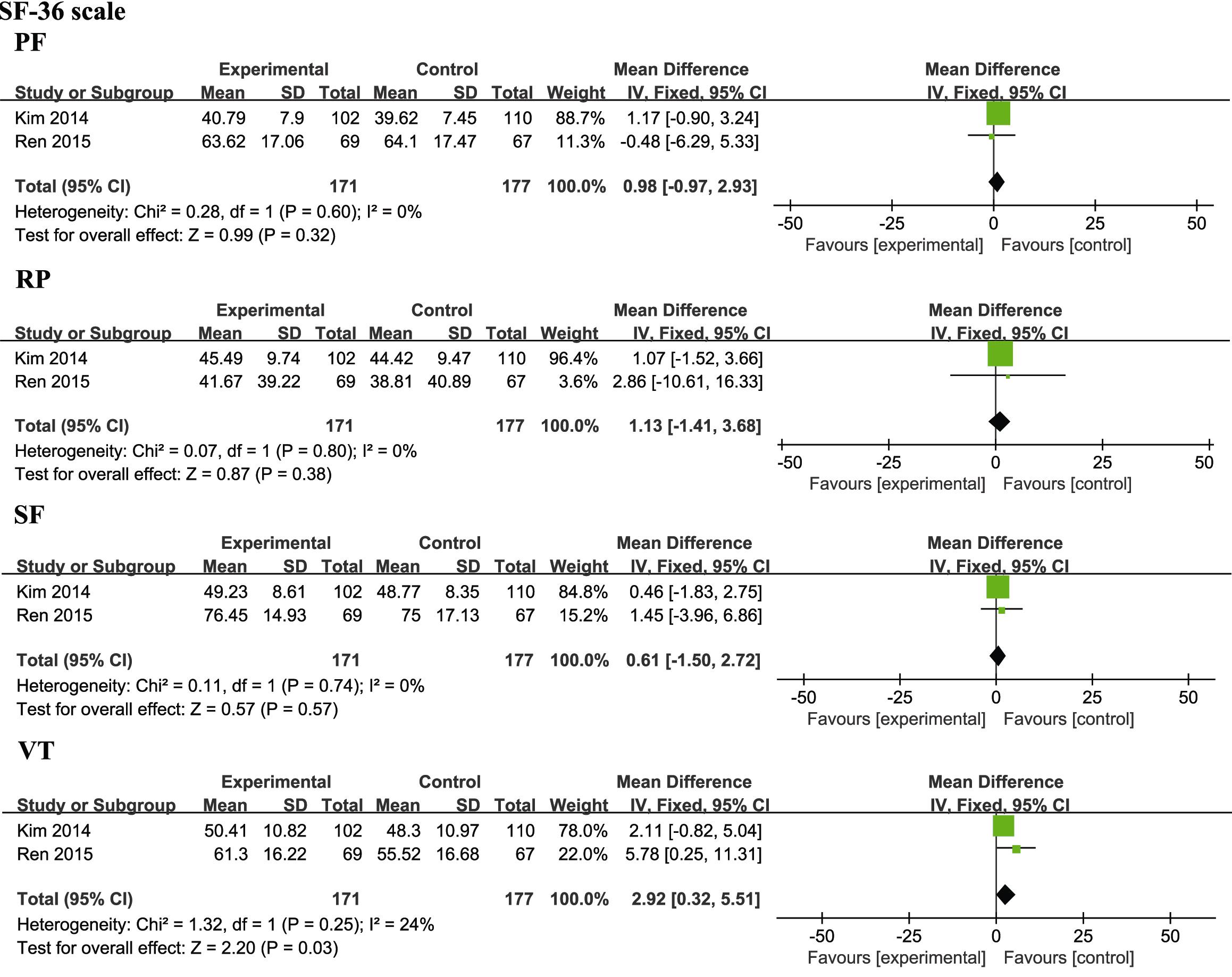

Supplement: Supplemental Digital Content [file medi-95-e3244-s001.doc]
